# Glioblastoma Stem-Like Cells (GSCs) with Mesenchymal Signature: Lipid Profiles of Mobile Lipids Obtained with MRS before and after Radio/Chemical Treatments

**DOI:** 10.3390/biom12081051

**Published:** 2022-07-28

**Authors:** Sveva Grande, Alessandra Palma, Anna Maria Luciani, Pasqualino Anello, Lucia Ricci-Vitiani, Mariachiara Buccarelli, Quintino Giorgio D’Alessandris, Roberto Pallini, Laura Guidoni, Vincenza Viti, Antonella Rosi

**Affiliations:** 1 National Centre for Innovative Technologies in Public Health, Istituto Superiore di Sanità, 00161 Rome, Italy; sveva.grande@iss.it (S.G.); alessandra.palma@iss.it (A.P.); annamaria.luciani@iss.it (A.M.L.); pasqualino.anello@iss.it (P.A.); laguidoni@gmail.com (L.G.); vincefl.viti@gmail.com (V.V.); 2 Department of Oncology and Molecular Medicine, Istituto Superiore di Sanità, 00161 Rome, Italy; lucia.riccivitiani@iss.it (L.R.-V.); mariachiara.buccarelli@iss.it (M.B.); 3 Institute of Neurosurgery, Fondazione IRCCS Policlinico Universitario A. Gemelli, 00168 Rome, Italy; giorgiodal@hotmail.it (Q.G.D.); roberto.pallini@unicatt.it (R.P.); 4 Institute of Neurosurgery, Università Cattolica del Sacro Cuore, 00168 Rome, Italy

**Keywords:** mesenchymal stem cells, glioblastoma, lipids, MR spectroscopy, unsaturation

## Abstract

Glioblastoma is the most common and lethal primary malignant brain tumor in adults. Glioblastoma stem cells (GSCs) promote and are responsible for glioblastoma intratumoral heterogeneity and therapy resistance, due to their two main features: self-renewal and differentiation. Lipids have important biological and physiological functions that are critical for understanding the regulation and control of stem cell fate; lipid metabolism and related unsaturation levels play a possible role as the target of therapeutics to overcome glioblastoma radioresistance. This paper aimed at an in-depth analysis of 13 GSC mesenchymal (MES) lines, two subclones, and a stabilized glioblastoma line (T98G) by magnetic resonance spectroscopy (MRS). Particularly, 2D MRS was used to investigate lipid unsaturation behavior during growth in culture and after treatment with etomoxir and photon beams. MES lines, although belonging to the same genetic and metabolic cluster, showed metabolic heterogeneity when observed by MRS, focusing on lipid signals. Nonetheless, the observed unsaturation level stability for two representative lines after stressful treatments suggests unusual robustness of the unsaturation levels for each line, as a peculiar and intrinsic characteristic of GSCs.

## 1. Introduction

Glioblastoma is the most common and lethal primary malignant brain tumor in adults; it is a heterogeneous tumor with multiple subclonal driver mutations (such as isocitrate dehydrogenase (IDH) mutation) that induce high adaptability and resistance to all therapeutic approaches [1,2,3]. For this reason, despite the multimodal standard treatments for glioblastoma, the median survival is still approximately one year [4]. It is well established that cancer cells adopt alternative metabolic pathways, and this metabolic reprogramming can lead to treatment resistance [5].

Moreover, glioblastomas are complex systems that rapidly evolve in response to severe environmental conditions and can coopt stem-like features to survive and progress [6]; they actively remodel their microenvironments through modulation of the immune system, stroma, and vasculature [2].

Glioblastoma stem-like cells (GSCs) reside in intratumoral hypoxic protective niches, where they are maintained in a slowly dividing state. They contribute, at least partly, to both glioblastoma intratumoral heterogeneity and resistance to pharmacology, radiation, and surgery, due to their two main features: self-renewal and differentiation [7,8,9]. GSCs recapitulate the heterogeneity of the parental tumor in vivo, and their biological relevance is demonstrated by their functional role in tumor growth and recurrence [10,11,12]; they can, therefore, be considered a key therapeutic target [11,12,13,14,15].

The high heterogeneity of GSCs has been demonstrated in previous studies. Lines from a collection of forty-four patient-derived GSCs have been assigned to two GSC clusters, one characterized by a proneural-like phenotype (PRO) and the other showing a mesenchymal-like phenotype (MES), by magnetic resonance spectroscopy (MRS) [16] gene expression profiling, and signal transduction pathway activation [17,18].

Particularly, GSC lines with a MES phenotype were characterized by high lipid content present in cells as lipid droplets (LDs), dynamic and structured cellular organelles [16]. Literature data report that MES stem-like cells could regulate the stemness of cancer stem cells by reconnecting lipid metabolism [19,20,21].

Lipids have important biological and physiological functions that are critical for understanding the regulation and control of stem cell fate. Lipid metabolism is also altered in rapidly proliferating cells and an increased lipogenesis has been considered as another metabolic hallmark of cancer cells [22,23]. Moreover, lipid metabolism reprogramming and dysregulation is recognized as an important factor in cancer metabolism [24]. Particularly, triglycerides (TG), the major components of LDs, have been demonstrated to serve as a critical energy reservoir to support glioblastoma cell survival [25]. Notwithstanding, the role played by lipid species, as the degree of FA unsaturation, is still a matter of debate [26].

Within the cell, FAs can be esterified with glycerol, leading to the formation of triglycerides which are then stored in LDs. LDs regulate the trafficking of polyunsaturated fatty acids (PUFAs) to prevent oxidative stress and cell death and the release of PUFAs for their conversion by cyclooxygenases and lipoxygenases into a whole range of oxygenated mediators of inflammation in cancer cells [27].

Glioma tissues have been shown to have enhanced lipid synthesis and MR studies have revealed a correlation between spectroscopic lipid signals from spectra of glioblastoma extracts and the grade of malignancy [28]. However, the mechanism by which lipids participate in the progression of brain tumors is still elusive, and there is little knowledge about the specific lipid composition of LDs in stem-like cells.

Mobile lipids (ML) in LDs are visible by MRS and are present as saturated, monounsaturated, and polyunsaturated FAs in TG molecules; MRS offers the unique possibility of examining properties of LDs in their intact environment. [29,30]. In particular, two-dimensional (2D) MRS may give important contributions to the study of glioblastoma lipid metabolism also in vivo [31].

The present paper aimed at an in-depth analysis by MRS of lipid metabolism and lipid unsaturation levels in 13 GSC MES lines plus two subclones and a stabilized glioblastoma line (T98G), to evidence their possible role as the target of therapeutics and in glioblastoma radioresistance. Given the importance of the eicosanoid cascade starting from arachidonic acid and the protective effects of omega-3 lipids, we analyzed the MR data of the MES subgroup of GSCs bound to high content of LDs in terms of 2D cross-peak intensity ratios, suggestive of different FA prevalence in the different lines, to obtain more insights into the unsaturation content of triglyceride molecules in LDs.

A possible link between lipid unsaturation levels and non-lipid metabolite concentrations was also investigated. Finally, changes in unsaturation level after treatment with external stressors etomoxir (an inhibitor of fatty acid oxidation (FAO)) and gamma radiation were studied in two MES lines, characterized by very high lipid content.

## 2. Materials and Methods

### 2.1. GSCs Isolation, Cell Culture

Tumor tissue samples were harvested from patients undergoing craniotomy at the Institute of Neurosurgery, Università Cattolica del Sacro Cuore (UCSC), Rome, Italy. All the patients provided written informed consent according to the research proposals approved by the Ethical Committee of UCSC. Patients were eligible for the study if a diagnosis of glioblastoma mutant for IDHas tested by mutational analysis of IDH1 and IDH2 genes [32] was established histologically according to the WHO classification [33]. GSC lines were isolated through mechanical dissociation of tumor tissues and cultured in a serum-free medium supplemented with EGF and basic FGF as described [34]. Details about patients’ treatments and GSCs isolation were given elsewhere and the neural origin and stemness of cultured cells was assessed by phenotypic and functional characterization [34,35]. Stem cell marker (CD133 and Sox2) expression was evaluated by flow cytometry with a Canto analyzer (Becton Dickinson, Milan, Italy). To assess clonogenicity, viable cells were seeded at different densities (1-3-10 cells/well) in 96-well plates by cell sorting (FACS Aria, Becton Dickinson). After two weeks, wells with growing clones were counted, and results were analyzed by extreme limiting dilution assay (ELDA) software. The in vivo tumorigenic potential of the GSC lines was assayed by intracranial or subcutaneous cell injection in immunocompromised mice. GSCs were capable of generating a tumor identical to the human tumor both in antigen expression and histological tissue organization. GSC lines were validated by short tandem repeat (STR) DNA fingerprinting. Cell proliferation was monitored by counting the cells and confirmed by using the CellTiter-Blue viability assay (Promega Italia srl, Milan, Italy). Clones from GSCs were obtained by plating single cells into 96-well plates. After 4 weeks, single clones were mechanically dissociated and replated to expand the culture.

### 2.2. ^1^H MRS Cell Sample Preparation

For MRS sample preparation, cells were removed and washed in PBS and centrifuged at 162 rcf for 3 min. The pellet was suspended in PBS with 20% D_2_O and 2mM sodium 3-(trimethylsilyl) propionate-2,2,3,3-d4 (TMSP) as a frequency standard. A 15 μL aliquot of the suspension was transferred into a 1 mm MR microtube and centrifuged to obtain a packed cell volume. All MRS reagents were purchased from Cambridge Isotope Laboratories, Inc. (Tewksbury, MA,USA).

All GSC MES lines samples were prepared and measured on the 4th day in culture. GSC line #74 samples were prepared and measured as a function of time in culture on the 4th, 7th, 10th, and 14th day in culture.

### 2.3. Etomoxir Treatment

For etomoxir treatments, 2000 cells (line #61 and line #74) in exponential growth phase at density of 2 × 10^4^ cells/mL were deposited in 75 cm^2^ flasks and incubated at 37 °C in a 5% CO_2_ atmosphere for MRS experiments. After one week of culture, cells were treated in triplicate with 200 μM Etomoxir (Sigma-Aldrich St. Louis, MO, USA) for 6 h. MRS experiments on control and treated samples were run 24 h later for both lines.

### 2.4. Irradiation Treatments

For irradiation treatments, line #61 and #74 cells were seeded as described previously and after 72 h, cells were irradiated in culture flasks at a single acute dose of 20 Gy with a cesium-137 gamma ray source (Gammacell 40 Exactor, NORDION, Ottawa, ON, Canada) operating at a dose rate of 0.66 Gy/min at Istituto Superiore di Sanità, Rome, Italy. A single radiation dose of 20 Gy was used, comparable to total doses delivered during radiation therapy in a fractionated regimen and to a single high dose used in special therapeutic modalities, such as intraoperative radiotherapy and stereotactic radiosurgery [36]. MRS experiments were run on control and irradiated samples 72 h after irradiation for both lines.

### 2.5. ^1^H MRS Measurements

^1^H MRS experiments were run on a digital Bruker Avance spectrometer at 400.14 MHz, equipped with a 1mm microprobe. Both one-dimensional (1D) and two-dimensional correlation spectroscopy (2D COSY) experiments were performed, at T = 298 K. The 1D ^1^H MRS spectra of GSCs were acquired with a 90° RF pulse, the number of scans (ns) was equal to 1000 (sufficient to achieve a good signal-to-noise ratio) for cell spectra while ns = 4000 was used for culture media spectra. When indicated, a Lorentzian–Gaussian function was applied in the time domain, before Fourier transformation. The 2D COSY spectra were acquired with a 90°–t1–90°–t2 pulse sequence and ns = 32 for cell or ns = 128 for culture media samples. Spectra were acquired as a matrix of 512 × 128 data points in time domain.

MRS parameters were obtained in at least three independent experiments and data expressed as mean ± standard deviation (SD) values. TopSpin 4.0.9 software (Bruker Biospin GmbH, Rheinstetten, Germany) was used to perform 2D cross-peak integration, as reported in [35]. The 2D signal integrals were normalized to the intensity of the lysine (Lys) cross peak at 1.70–3.00 ppm. This peak was considered representative of the cellular mass, as it was found to be constant in a number of cell models and tissue samples [35].

### 2.6. Statistical Analysis

Unsupervised agglomerative hierarchical clustering, principal component analysis, and Student’s t test were performed utilizing PAST 4.03 Software, version 2020 (https://past.en.lo4d.com/windows, accessed on 15 July 2022) [37].

## 3. Results

### 3.1. Lipid Metabolism in MES GSCs Lines

**Figure 1:**
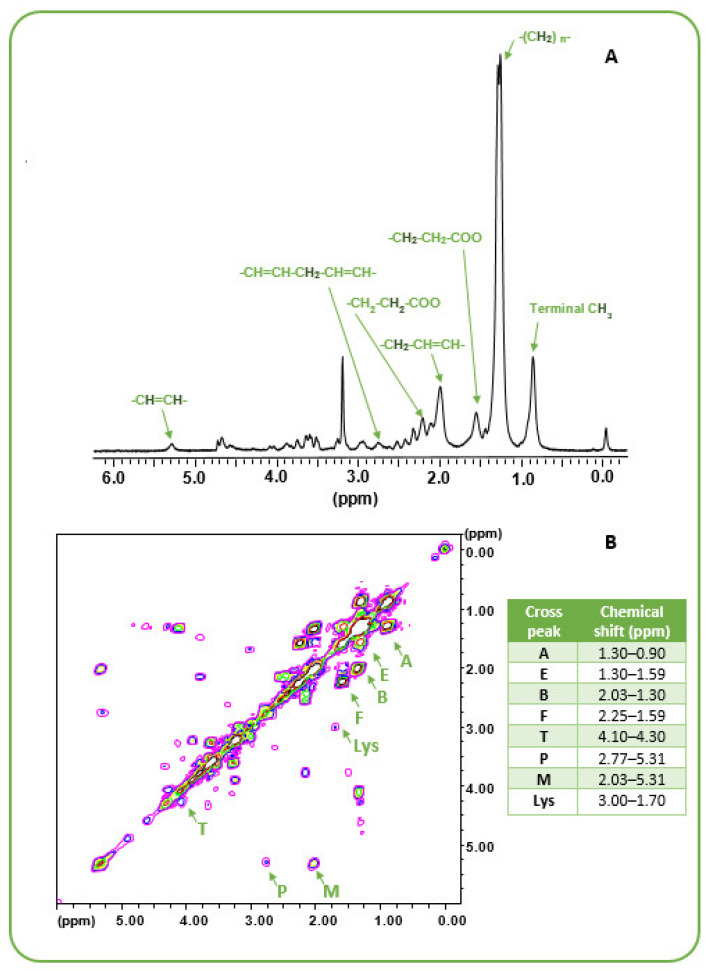
(**A**) The ^1^H MR spectrum of GSC line #74 belonging to the MES cluster chosen as representative of the analyzed cell lines. Signals of interest from the different chemical groups in FA chains are labeled according to Peterson et al. 2020 [38]. (**B**) The 2D MR COSY spectrum of line #74. Main lipid cross peaks analyzed in this study are labeled together with the lysine (Lys) cross peak used as an internal reference for the intensities. The Table reports the corresponding chemical shifts.

Typical MR 1D MRS and 2D-COSY spectra from representative MES cell line #74 are shown in Figure 1A,B, respectively, where main lipid peaks are labeled according to the previous literature [35,38,39]; their chemical shifts are reported in the enclosed table.

Supplementary Table S1A reports the description of the different fatty acid features with the connectivity giving rise to 2D MRS acyl chain cross-peaks (A, A’, B, B’, E, E’, F, F’, M, M’, and P), while Figure S1 shows the structure of a linoleic acid molecule as representative of the class of lipids for peak assignment. Peaks A’, B’, E’, F’, and M’ were not detectable in our spectra.

Moreover, M/A and P/A ratios are related to mono- and polyunsaturation, respectively (Table S1A,B). Table S1B reports cross-peak intensity ratios for the widely present fatty acids in cells. B/A, F/A, E/A, F/B, B/M ratios are reported as indicative of the contribution to MR visible cross peaks of the different fatty acids.

We have examined intensities of these lipid signals from spectra of 13 MES lines, two subclones with MES signature, and one commercial glioblastoma line, namely T98G. Clinical parameters, patient demographics, and features of the 13 MES lines are reported in Supplementary Table S2.

**Figure 2:**
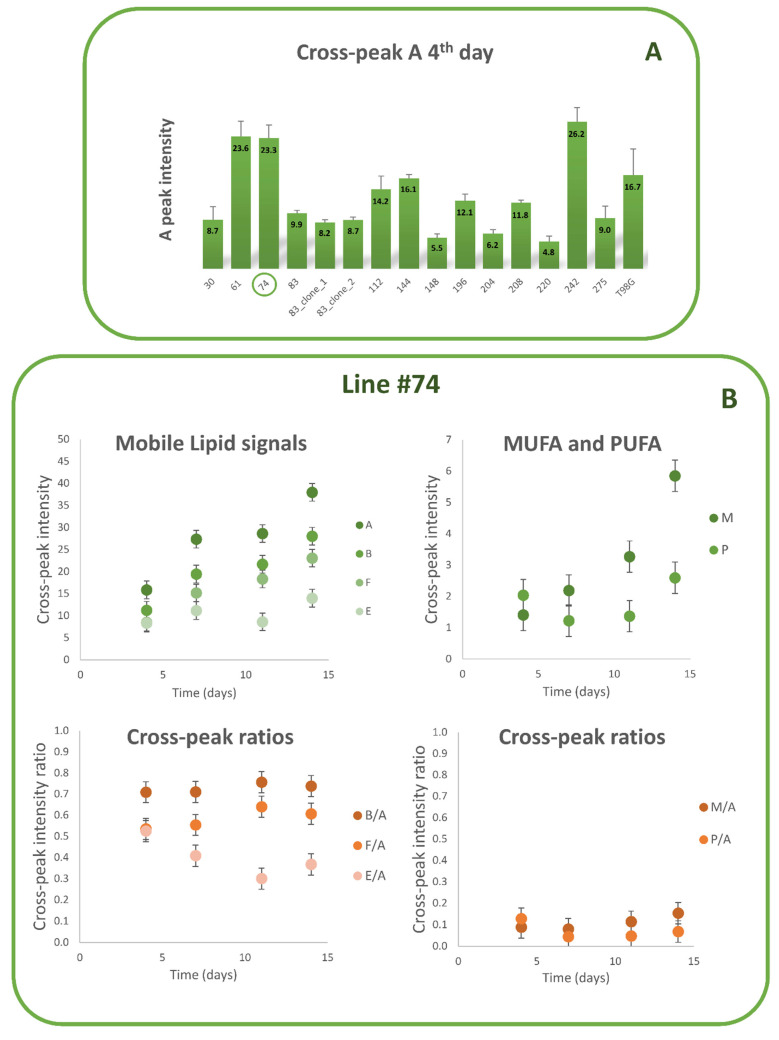
(**A**) Intensity of the lipid cross peak A calculated on the 4th day after seeding for all 13 GSCs lines with MES profile, together with two clones from line #83 and the stabilized tumor line T98G. (**B**) Intensities of cross peaks A, B, E, F, M, and P from line #74 spectra (upper graphs) and ratios of the same cross peaks with respect to cross peak A (lower graphs) are reported as a function of time during cell growth up to 14 days after seeding. Each point is the average of at least three independent experiments; standard deviations were assigned as error bars.

The intensity of the cross peak A calculated on the 4th day in culture is reported in Figure 2A for all examined lines and clones. Intensities of cross peaks A, B, F, E, M, and P and ratios B/A, F/A, E/A, M/A, and P/A from GSC line #74 spectra, as a function of time in culture, are shown in Figure 2B. GSC #74 was selected among MES lines with a higher lipid content (Figure 2A).

Intensity of all mobile lipid (ML) signals increased during cell growth as a function of time in culture, as previously observed in GSCs [35,40,41], as well as in tumor cell lines [42], while their ratios were almost constant during growth (Figure 2B).

**Figure 3:**
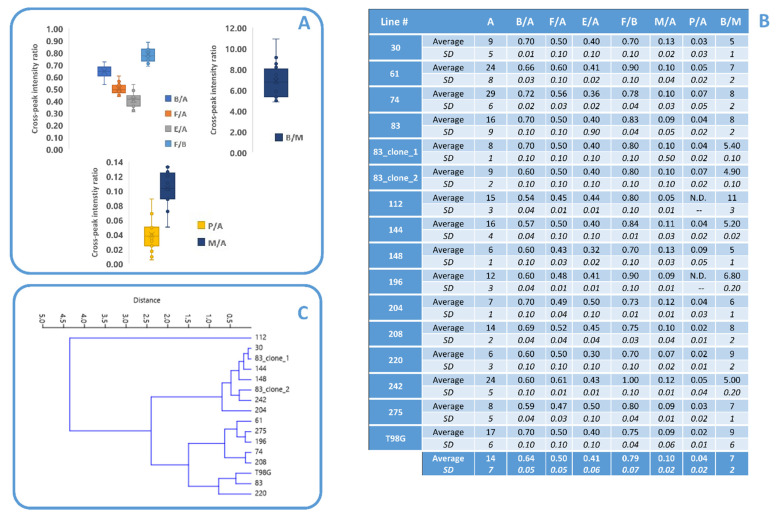
(**A**) Box and Whisker Plot comparing the cross-peak ratios B/A, F/A, E/A, F/B, B/M, M/A, and P/A from all MES cell line spectra. (**B**) Average values and standard deviations of the aforementioned ratios and of cross peak A for each MES cell line, for the subclones, and T98G, and for all analyzed ones calculated in at least three independent experiments. Average and standard deviation inter lines for each parameter is also reported. (**C**) Dendrogram resulting from unsupervised cluster analysis of values of eight metabolic parameters related to lipid unsaturation from MR spectra from all cell lines (B/A, F/A, E/A, M/A, P/A, (M+P)/A, F/B, B/M). All these MES cell lines are grouped into a single cluster with the only exception of GSC line #112. N.D. stands for undetectable.

Average values of ratios B/A, F/A, E/A, F/B, B/M, P/A, and M/A for all examined lines monitored between the 4th and 14th day after seeding are reported in a Box and Whisker Plot (Figure 3A) and the enclosed table (Figure 3B). The values of cross-peak ratios reported in Table B of Figure 3 for all analyzed lines evidenced that, although a homogeneity of intensity ratios could be envisaged by the presented data, a few lines differed for some ratios. In fact, while all lines were characterized by the presence of signals from monounsaturated lipids, polyunsaturated ones were undetectable in lines #112 and #196, while line #220 was characterized by the lowest sum of P/A and M/A ratios (Table B in Figure 3). Line #112 also showed a low value of B/A ratio. Lines #112 and #220 were also characterized by the highest value of B/M.

To have more insights on the anomalous behavior of the listed cell lines, an unsupervised cluster analysis was performed by taking into account all lipid signals evidencing an anomalous behavior only for line #112 (Figure 3C).

### 3.2. Comparison between Lipid and No Lipid Metabolism

To obtain more insight into the meaning of lipid unsaturation in these cells, we examined if a link existed between the lipid unsaturation level and that of other metabolites.

**Figure 4:**
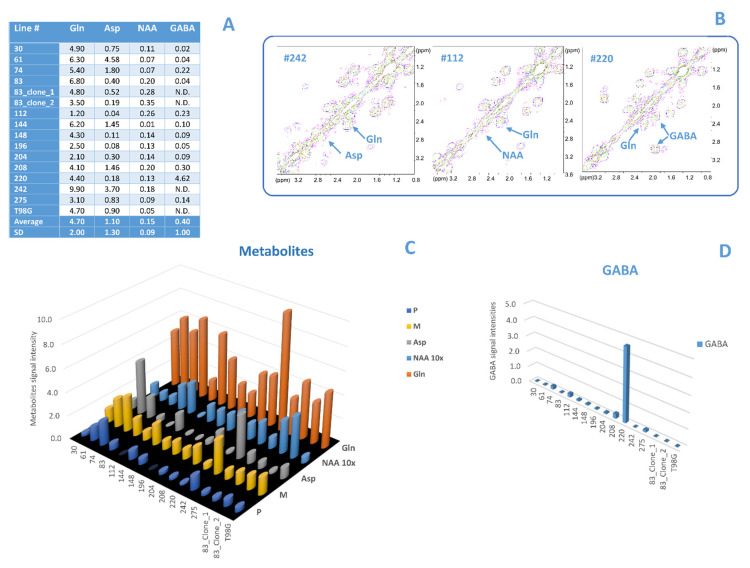
(**A**) Average values and standard deviations of all analyzed no lipid metabolites (Gln, Asp, NAA, GABA) signal intensities, calculated on at least three independent experiments. (**B**) 2D COSY spectra of lines #242, #112, and #220, respectively; regions of interest are shown: Gln cross peak at 2.44–2.13 ppm, Asp cross peak at 2.80–2.68 ppm, NAA cross peak at 2.67–2.48 ppm, and GABA cross peak at 2.30–1.90 ppm. (**C**,**D**) Bar graphs reporting cross-peak intensities of Gln, Asp, NAA, M, and P (**C**), and GABA (**D**) for all analyzed MES cell lines, for the subclones and T98G. The intensity of the NAA cross peak is reported as 10×. N.D. stands for undetectable.

Given that the literature reported a strict relation of low aspartate (Asp) and glutamine (Gln) levels with the proliferative status of cells [43,44], we examined intensities of these metabolites and correlated them with lipid signal intensity. By comparing the tables in Figure 3B and Figure 4A, lines #112, #275, #196 showed both low levels of Asp, Gln and unsaturation. On the other hand, line #112 presented a value higher than average of the neuronal marker N-acetylaspartate (NAA) (Figure 4A–C), line #242 high values of Asp and Gln (Figure 4A–C), and line #220 an anomalous value of the marker γ-aminobutyric acid (GABA) (Figure 4A,B,D). No anomalous behaviors were observed with other metabolites such as choline, phosphocholine, glycerylphosphorylcholine, N-acetylgalactosamine, uridine diphosphate glucose (data not shown).

### 3.3. Etomoxir and Irradiation Treatment

**Figure 5:**
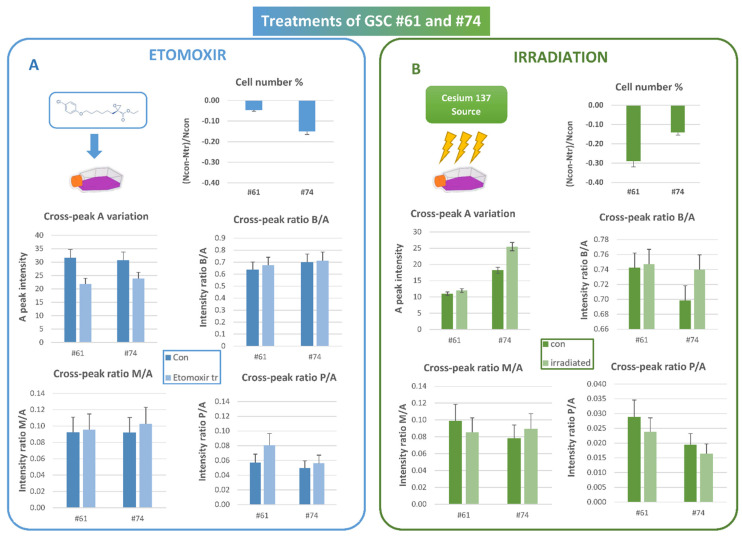
(**A**) Effects of etomoxir treatment on cell number, cross peak A intensity and cross peak B/A, M/A, and P/A ratios measured 24 h after treatment of cell lines #61 and #74. Each point is the average of at least three independent experiments; standard deviations were assigned as error bars. (**B**) Effects of photon irradiation on cell number, cross peak A intensity and cross peak B/A, M/A, and P/A ratios measured 72 h after irradiation of cell lines #61 and #74. Each point is the average of at least three independent experiments; standard deviations were assigned as error bars.

To highlight the unsaturation level sensitivity to external stressors, we examined the response to chemical and radiation treatments of two MES lines, namely lines #61 and #74, among those of cell groups here studied with higher lipid content. Both cell lines have been treated with a sublethal dose of etomoxir and observed after 24 h. A cytotoxic effect was observed only for line #74 (Figure 5A). After treatment, the A signal intensity decreased in both lines, while unsaturation was slightly affected or almost unaffected, as indicated by constant values of ratios B/A, M/A, and P/A (Figure 5A).

The two lines were then treated with photon beams with a dose of 20 Gy (Figure 5B). The cytotoxic effect induced by photon beams on both lines 72 h after irradiation is reported (Figure 5B). Cross peak A signal intensity and the ratio B/A increased to a high extent at 72 h after irradiation only in line #74, remaining almost unaffected in line #61 (Figure 5B). On the other hand, no significant changes for M/A and P/A ratios were observed for both lines after irradiation (Figure 5B).

## 4. Discussion

Mesenchymal stem cells (MSCs) are a type of multipotent stem cell that plays important roles in regeneration and wound healing [19]. In the tumor microenvironment (TME), MSCs undergo transformation to support the cancer cell growth, thus promoting cancer progression [20,21,45,46]. Literature data report that MSCs could regulate the stemness of cancer stem cells (CSCs) by reconnecting lipid metabolism [47,48,49]. The upregulation of intracellular lipid metabolism, except for lipid peroxidation, plays important roles in maintaining the stemness of CSCs and promoting the growth and progression of cancer, and has been correlated with poor prognosis [50,51].

Metabolic targeting has long been advocated as a therapy against many tumors including glioblastoma, but how lipid metabolism is altered to suit different microenvironmental conditions and whether CSCs have altered lipid metabolism are still yet to be solved [52,53]. FAs play the role of an alternative fuel pathway for CSCs; the elevated FAO can maintain CSCs self-renewal by modulating lipid and membrane synthesis, quenching ROS through NADPH production, and promoting chemoresistance [54]. A lipogenic switch has been observed in CSCs, which facilitate the production of monounsaturated lipids that are less susceptible to lipid peroxidation, thus modulating the detrimental effect of ROS, facilitating cancer progression and metastasis [54,55,56].

In previous studies, GSCs lines have been classified according with their metabolic and genomic profiles as PRO and MES [16,17,18]. Particularly, high ML signals have emerged as a characteristic of MES GSC lines [16], being correlated to the presence of LDs; increased abundance of LDs is a feature of many aggressive cancers [57,58,59,60].

In the present work, we tried to quantify by 2D MRS the unsaturation content of TG molecules in LDs from 13 different MES GSCs and observed their behavior during cell growth in culture and after treatment with etomoxir and radiation. In fact, 2D MRS is a powerful tool to investigate the FA chemical composition of the TG, particularly the fraction of saturated, monosaturated, and polyunsaturated fatty acids, allowing a better resolution of metabolic signals that are overlapping in 1D spectra [30]; ratios of the intensities of the FA-derived 2D COSY cross peaks can be easily measured [31].

The lipid in LD concentration, measured as A, B, E, and F peak intensity, together with MUFA (M) and PUFA (P) signals, increase as a function of days in culture, in agreement with previous work [40,41]. On the other hand, cross-peak ratios B/A, E/A, and F/A, indicative of lipid unsaturation to different extents (see Tables S1 and S2), and M/A and P/A ratios, indicative of mono- and poly-unsaturation, respectively, remains almost constant in the same temporal window. It has been reported that rapidly proliferating cancer cells have a greater demand for MUFAs, which are utilized mainly for the synthesis of membrane PLs and TAGs, and indeed many cancer cells are characterized by a higher relative proportion of MUFAs than corresponding normal tissues [61]. The MES “lipid accumulating” phenotype, may enable cells to make use of lipid stores in conditions of stress or limiting access to lipids. It is, then, likely that the balance between these two families (MUFA and PUFA) of desaturated lipids have a profound impact on membrane properties and therapy response/resistance of cancer cells.

On the other hand, cross-peak ratios B/A, E/A, F/A, M/A, and P/A seem likely not to be influenced by culture conditions, as hypoxia induced by increased cell density, thus suggesting they represent a specific feature of the cell line mainly related to the different FA composition.

Analysis of the FA cross-peak ratios suggests differences in PUFA, in omega-3, and in omega-6 levels in these patient-derived cells, though comprised in the same MES group. Furthermore, parameters of subclones of MES line #83 and of T98G with similar lipid features fit the distribution of the MES lines.

Recent studies suggest a role of omega-3 in prevention and therapy of brain cancer, as omega-3 and omega-6 play a role in brain health [62]. PUFAs are necessary for normal physiological events in the nervous system and alterations in PUFA signalling are involved in the development of several diseases of the nervous system and of cancer. With this respect, monitoring of changes in the equilibrium between different PUFAs may give information that is hopefully useful for patient treatment.

One recent newsworthy question is the role played by desaturation in tumor cell stemness. Besides FAO, lipid desaturation to modify FA chain seems implicated in CSC regulation in many kinds of tumors [63]. Stearoyl coenzyme A desaturase 1 (SCD1), a key enzyme found in endoplasmic reticulum (ER) responsible for the conversion of saturated FAs to unsaturated Fas, is crucial for protecting the cell from lipotoxicity and it is necessary for the proliferation of several malignancies [64,65,66,67]. Recently, it was shown that GSCs depend on the adaptive activation of ER stress and subsequent activation of lipogenesis and particularly of SCD1, which promotes ER homeostasis, cytoprotection, and tumor initiation [68]. The high level of saturated FAs in LDs of analyzed MES lines could be related to this promotion of tumor survival under ER stress.

### 4.1. Comparison between Lipid and No Lipid Metabolism

Although unsaturation levels are similar in all examined lines, it is worth noting that significantly lower levels in some GSC MES lines can be related to anomalous intensity values of no lipid metabolites. In fact, line #220, that presented low values of B/A and sum of P/A and M/A, was characterized by a very high value of the neurotransmitter GABA signal, while in the other lines GABA signals were barely detectable. Furthermore, this line was characterized by the lowest value of A peak intensity even on the 14th day in culture (not shown). Both GABA and A signal intensity are similar to those observed for PRO cluster lines [16], suggesting an anomalous behavior of this line.

A different explanation could be hypothesized for the low unsaturation level observed in lines #112 and #196, as well as for line #275 to a minor extent. The absence of P signal in the spectra of lines #112 and #196 and of relative ratios for these two lines could be a consequence of a high presence of saturated oleic chains (Table S1A,B). On the other hand, line #112 showed a mismatch comparing genetic and metabolic clustering in the past [17].

On the opposite side, lines #148 and #242 were characterized by the highest values of both M/A and P/A together with low values of B/A ratio; this may derive from a consistent presence of EPA and DHA (Table S1A,B). Additionally, in this case, the anomalous behavior of line #242 confirms the mismatch within genetic and metabolic signature observed in previous work [18].

Moreover, in line #112, low levels of unsaturation were concomitant to low aspartate and glutamine concentration. Both types of metabolism are closely involved in glioblastoma chemoresistance. The literature reported a relationship between low Asp and Gln levels and the proliferative status of cells and cellular redox homeostasis; in particular, insufficient cytosolic aspartate delivery leads to cell death when TCA cycle carbon is reduced following glutamine withdrawal and/or glutaminase inhibition [43,44]. In fact, most proliferating cells in culture depend on two main carbon sources: glucose and glutamine [69]. Initiating de novo lipogenesis in mitochondria through citrate production is also vital for proliferation in some contexts [70,71]. The low levels of unsaturation, Gln and Asp in line #112 could, therefore, indicate an anomalous proliferative status of this line.

### 4.2. Irradiation and Etomoxir Treatment

In previous studies, we observed the response of GSCs to drug and radiation treatments, addressing mainly the energetic metabolism and, indirectly, the lipid and glutamine involvement as energy sources. Particularly, oligomycin, a mitochondrial ATPase inhibitor, induced high lactate extrusion, producing neutral lipid accumulation [16]. Moreover, an increase in lipid concentration was observed in the PRO line #1 after treatment with radiation of different qualities associated to cytotoxic effects, while for the MES line #83, only cytostatic effects were observed, paralleled by statistically non-significant effects on lipid signal intensity [40,41]. The differences in radiation response may be likely attributed to genetic and metabolic differences between the MES and PRO GSC lines [40].

The present work added further information to what was observed in the past. An increase in mobile lipid cross peak A and B/A signal ratio after irradiation, paralleled by cytotoxic effects, was observed only in line #74. Therefore, this allows us to infer that effects due to radiation are different in different lines, and even if lines #61 and #74 belong to MES clusters, their response to stressors are different, with the behavior of line #74 more closely resembling what was observed in PRO line #1 [40,41]. 

On the other hand, etomoxir, a widely used small-molecule inhibitor of FAO through its irreversible inhibitory effects on CPT1a, induced a cytotoxic effect in both MES lines, more evident in line #74, associated to a decrease in lipid content measured as cross peak A, as expected. This is in agreement with a recent study, in which lowered glycolysis and a remarkably low level of cytoplasmic lipids were detected in radioresistant glioblastoma cells after FAO inhibition by etomoxir [72]. The level of unsaturation (cross-peak ratios B/A, M/A, and P/A) seems not to be affected by either kind of treatments.

These data may indicate that lipid storage may or may not be used as cell fuel for membrane synthesis, concomitant to a proliferative arrest induced in these lines. This behavior may not be, therefore, attributable to the MES profile of these GSCs, as they behave differently: the contribution of membrane lipid changes to oncogenic signaling appears to be complex and multifactorial.

## 5. Conclusions

Analysis of the FA cross-peak ratios allowed us to detect differences in unsaturation levels in patient-derived GSCs, even if belonging to the same MES group: monitoring of changes in the equilibrium between different PUFAs may give information that is hopefully useful for patient treatment. The high level of saturated FAs in LDs of the analyzed MES lines could be related to the promotion of tumor survival under stress conditions. However, a definite explanation cannot be drawn by present results: probably, different contributions can be ascribed.

The concomitance of low levels of unsaturation with low aspartate and glutamine concentration may play a relevant role, with both types of metabolism closely involved in glioblastoma chemoresistance; in our experiments the low levels of unsaturation, Gln, and Asp in line #112 could, therefore, indicate an anomaly of this line and deserves future investigations in terms of response to therapy.

Finally, the unsaturation level stability in all observed lines either after etomoxir and irradiation treatment suggest a robustness of the unsaturation level of the GSC lines to be likely considered as a specific feature of the cell line mainly related to different FA composition.

These results contribute to the rapidly growing interest in lipid targeting in cancer that represents a central hallmark of cancer with a promising strategy for clinical application. The link with diet, including dietary lipids, will also create unique opportunities for preventive strategies and therapy enhancement, especially for personalized medicine.

## Data Availability

All the data and materials used and/or analyzed during this study are available from the corresponding authors on reasonable request.

## Supplementary Materials

The following are available online at https://www.mdpi.com/article/10.3390/biom12081051/s1; Table S1: Fatty acid features, Figure S1: Structure of a linoleic acid molecule and MRS parameters, Table S2: Patient demographics, clinical information and glioblastoma features.
