# Supplementary material for: Glioblastoma Stem-Like Cells (GSCs) with Mesenchymal Signature: Lipid Profiles of Mobile Lipids Obtained with MRS before and after Radio/Chemical Treatments
Source: Biomolecules. 2022 Jul 28;12(8):1051. doi: 10.3390/biom12081051 (PMC9405836; doi:10.3390/biom12081051)
Supplement: Supplementary file 1 [file biomolecules-12-01051-s001.zip › biomolecules-1776087-supplementary.pdf]

Supplementary material

Table S1. Fatty Acid features

(**A**) Chain length; ω nomenclature - number of carbons from the methyl end (ω end) to the first carbon in the double bond closest to the methyl end-; main lipid cross peaks observed in 2D COSY MR spectra and their numerousness, for the widely present fatty acids in cells (EPA: Eicosapentaenoic acid; DHA: Docosahexaenoic acid). When protons usually found as A, B, E and F are in different chemical coupling state, such as in ω-3, they shift to other 2D positions (A', B', E' and F') being below the detection limit of the experiments.

(**B**) Levels of unsaturation, expressed as cross peak intensity ratios for the widely present fatty acids in cells. B/A, F/A, E/A, F/B, B/M ratios are indicative of total lipid unsaturation while M/A and P/A ratios of mono- and polyunsaturation, respectively.

Table S1 A

Fatty acid	Chain length	Unsaturation ω	Cross Peaks
Stearic acid	18:0		A-F-E
Oleic acid	18:1	(ω-9)	A-2B-F-E-2M
Linoleic acid	18:2	(ω-6)	A-2B-F-E-2M-2P
α-linolenic acid	18:3	(ω-3)	A'-B-F-E-2M-4P
γ-linolenic acid	18:3	(ω-6)	A-2B-F-E-2M-4P
Arachidonic acid	20:4	(ω-6)	A-B-B'-F'-2M-6P
EPA	20:5	(ω-3)	A'-B'-F'-2M-8P
DHA	22:6	(ω-3)	A'-E'-F'-M-M'-10P

Table S1 B

Fatty Acid	B/A	F/A	E/A	F/B	M/A	P/A	B/M
Stearic acid	ØB,1A	1F,1A	1E,1A	1F, ØB	ØM,1A	ØP,1A	ØB,ØM
Oleic acid	2B,1A	1F,1A	1E,1A	1F,2B	2M,1A	ØP,1A	2B,2M
Linoleic acid	2B,1A	1F,1A	1E,1A	1F,2B	2M,1A	2P,1A	2B,2M
α-linolenic acid	1B, ØA	1F, ØA	1E, ØA	1F,1B	2M, ØA	4P, ØA	1B,2M
γ-linolenic acid	2B,1A	1F,1A	1E,1A	1F,2B	2M,1A	4P,1A	2B,2M
Arachidonic acid	1B,1A	ØF,1A	ØE,1A	ØF,1B	2M,1A	6P,1A	1B,2M
EPA	ØB, ØA	ØF, ØA	ØE, ØA	ØF, ØB	2M, ØA	8P,ØA	ØB,2M
DHA	ØB, ØA	ØF, ØA	ØE, ØA	ØF, ØA	1M, ØA	10P,ØA	ØB,1M

Figure S1. Structure of a linoleic acid molecule and MRS parameters

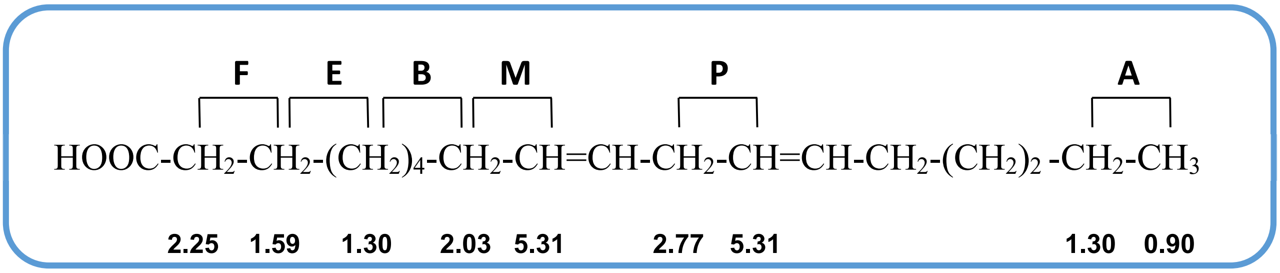

Structure of a molecule of linoleic acid, together with the connectivities giving rise to 2D COSY MR cross peaks (labelled) and the corresponding chemical shifts in the spectrum.

Table S2. Patient demographics, clinical information and GBM features.

Clinical parameters, patient's demographics and features of the 13 GSC lines, all deriving from tumors localized close to the temporal sub-ventricular zone.

GSC Line #	Age	Sex	previous chemo/RT	Up-front/ recurrent	IDH	Core/Periphery
#30	44	M	no	Up-front	wildtype	core
#61	59	M	no	Up-front	wildtype	core
#74	70	F	no	Up-front	wildtype	core
#83	52	M	no	Up-front	wildtype	core
#112	49	F	no	Up-front	wildtype	core
#144	57	M	no	Up-front	wildtype	core
#148	55	M	yes	recurrent	wildtype	core
#196	71	F	no	Up-front	wildtype	core
#204	80	F	no	Up-front	wildtype	core
#208	68	M	yes	recurrent	wildtype	core
#220	62	M	no	Up-front	wildtype	core
#242	64	M	no	Up-front	wildtype	core
#275	58	M	no	Up-front	wildtype	core
